# Variation in Salamander Tail Regeneration Is Associated with Genetic Factors That Determine Tail Morphology

**DOI:** 10.1371/journal.pone.0067274

**Published:** 2013-07-03

**Authors:** Gareth J. Voss, D. Kevin Kump, John A. Walker, S. Randal Voss

**Affiliations:** 1 Department of Biology & Spinal Cord and Brain Injury Research Center, University of Kentucky, Lexington, Kentucky, United States of America; 2 Math, Science, and Technology Center, Paul Laurence Dunbar High School, Lexington, Kentucky, United States of America; University of Sheffield, United Kingdom

## Abstract

Very little is known about the factors that cause variation in regenerative potential within and between species. Here, we used a genetic approach to identify heritable genetic factors that explain variation in tail regenerative outgrowth. A hybrid ambystomatid salamander (*Ambystoma mexicanum* x *A. andersoni*) was crossed to an *A. mexicanum* and 217 offspring were induced to undergo metamorphosis and attain terrestrial adult morphology using thyroid hormone. Following metamorphosis, each salamander’s tail tip was amputated and allowed to regenerate, and then amputated a second time and allowed to regenerate. Also, DNA was isolated from all individuals and genotypes were determined for 187 molecular markers distributed throughout the genome. The area of tissue that regenerated after the first and second amputations was highly positively correlated across males and females. Males presented wider tails and regenerated more tail tissue during both episodes of regeneration. Approximately 66–68% of the variation in regenerative outgrowth was explained by tail width, while tail length and genetic sex did not explain a significant amount of variation. A small effect QTL was identified as having a sex-independent effect on tail regeneration, but this QTL was only identified for the first episode of regeneration. Several molecular markers significantly affected regenerative outgrowth during both episodes of regeneration, but the effect sizes were small (<4%) and correlated with tail width. The results show that *ambysex* and minor effect QTL explain variation in adult tail morphology and importantly, tail width. In turn, tail width at the amputation plane largely determines the rate of regenerative outgrowth. Because amputations in this study were made at approximately the same position of the tail, our results resolve an outstanding question in regenerative biology: regenerative outgrowth positively co-varies as a function of tail width at the amputation site.

## Introduction

When considering the regenerative potential of vertebrate organisms, salamanders are noted for their unrivaled ability to regenerate multiple body parts while mammals are regarded as regeneration limited [1]. However, in reality, regenerative potential varies among species of a taxonomic group and probably also among individuals of a population. For example, some related adult salamanders of the genus *Ambystoma* regenerate limbs very quickly while others regenerate slowly [2], [3]. It has also been reported that some salamanders lose the ability to regenerate later in life, especially after metamorphosis, and that a few species have lost the ability to regenerate limbs altogether [4]. A striking example of regeneration variation is seen between normal mice and super-healer, MRL mice that are capable of healing ear punch wounds via a regenerative response that does not include scar formation [5]. The healing response depends upon the action of at least 17 quantitative trait loci (QTL), some of which appear to be associated with genetic sex and *p21,* a cell-cycle regulatory gene [6]–[10]. More recently, Seifert et al. [11] showed that African spiny mice (*Acomys*) autotomize skin presumably as a mechanism to elude predators. After autotomy, skin and hair regenerate without scarring. These observations suggest that regeneration varies within and between closely related species. Studying the basis of this variation presents an approach to identify factors that are associated with regenerative potential [12].

Salamanders perfectly regrow their tails after amputation or bite injuries [13]. Within hours after tail injury, epidermal cells cover the wound surface and innate immune responses are activated. During the first week of repair, a wound healing phase transitions into a cell proliferation phase as blastemal cells organize and increase in number. The tail steadily elongates over the next few weeks as tissues reform in the wake of the outgrowing, blastema cell population. The tail encodes positional information so that only the missing part is regenerated, and in the newt (*Notophthalamus viridescens*), the rate of regeneration is faster for more proximal amputation injuries [14], [15]. This has the effect of normalizing the amount of time for regeneration to complete along the proximal-distal axis. However, exactly how positional information and regeneration rate are determined remain outstanding questions in regenerative biology. The developmental stages of tail regeneration appear to be conserved among salamander species that present different tail morphologies; for example, between terrestrial adult newts that have relatively thin tails and aquatic Mexican axolotls (*Ambystoma mexicanum*) that have wide, keeled shaped tailfins adapted for swimming. Presumably, regeneration is also conserved between male and female salamanders of species that present sexually dimorphic tail morphologies, however the effect of genetic sex on tail regeneration is unknown.

We have shown previously that it is possible to cross *A. mexicanum* to related species and use linkage analysis to locate genetic factors that are associated with complex phenotypes [16]–[18]. This suggests that alleles with different phenotypic effects on regeneration may be fixed among related *Ambystoma* species. To test this hypothesis, we made an interspecific backcross using *A. mexicanum* and *A. andersoni,* and then performed genetic and morphological analyses to identify factors associated with variation in tail morphology and regeneration. We report that genetic factors, including the sex-determining locus (*ambysex*), explain variation in tail length and width. In turn, tail width at the injury site explains the majority of variation in regenerative outgrowth.

## Materials and Methods

### Ethics Statement

Methods for animal care and use followed recommendations in the Guide for the Care and Use of Laboratory Animals (Office of Laboratory Animal Welfare), and were approved by the University of Kentucky, Animal Care and Use Committee (Protocol # 00907L2005).

### Genetic Crosses

In this experiment we used offspring from a backcross (AxAn1) between an *A. mexicanum/A. andersoni* F1 hybrid and *A. mexicanum* [18]. These salamanders were induced to undergo metamorphosis at 120 days post fertilization (dpf). At 300 dpf, 215 metamorphs were anesthetized in 0.02% neutral buffered-benzocaine and 10% of the total tail length (measured from posterior vent to tail tip, approximately 1.0–1.2 cM) was removed from the caudal end of each salamander’s tail using a sterile razor blade. The tail tips were retained for DNA extraction and genotyping. The salamanders were reared for 51 days and then the tip of the tail was amputated following the procedure described above. The amount of tail removed was 1 cm proximal to the first amputation plane. The tips from each salamander were placed into separate glass vials containing formalin. Salamanders were revived and reared for 46 additional days and then at 400 dpf, tail tips were collected as described above and body weight was measured. Most of the individuals from the experiment were euthanized and sex was determined by visual inspection of gonads within the body cavity. For individuals that were not euthanized, sex was determined from cloaca size and shape. We determined retrospectively from necropsies that cloaca size and shape is 100% reliable for predicting the sex of terrestrial individuals from this cross.

### Preparation and Analysis of Tail Tips

Tail tips were subsequently processed through a series of solutions to clear pigments and fix tissues. First, tail tips were placed in two changes of water over two days to remove formalin. Next, they were placed in a solution of dilute hydrogen peroxide to remove pigment. Then, they were immersed in two changes each of 100%, 90%, 75%, and 50% ethanol. Finally, they were placed in methyl salicylate for permanent storage. Fixed tail tips were imaged while immersed in methyl salicylate and against a 100-micron scale grid. Images were taken using an Olympus SZX12 with a 0.5x objective lens and Olympus CCD Magnifire camera. The images were subsequently analyzed using NIH’s freeware program *ImageJ*. A line was drawn along the amputation plane, which was discernible due to a change in coloration and shape where the tail tip was surgically removed prior to regeneration. The area of tissue caudal to this amputation plane presented the amount of tissue regenerated. A parallel line was then created and positioned 4 mm rostral to the amputation plane. The area between these two parallel lines, and bounded by the dorsal and ventral periphery of the tail, provided an estimate of salamander tail width adjacent to the amputation plane. Area measurements were obtained using the polygon tool. Linear statistical models were formulated and SAS JMP 10.0 (SAS Institute, Cary, NC) was used to test the effects of sex, molecular markers, and morphology on tail tip regeneration.

### Genotyping, Linkage Map Construction, and QTL Mapping

DNA was isolated from tail-tip tissue of 217 AxAn1 individuals and these were used as template to obtain molecular marker genotypes as described in Voss et al. [18]. Marker orders were determined using MultiPoint 2.2 (MultiQTL Ltd., Hafia, Israel) and the Kosambi [19] mapping function. QTL were identified using R/qtl [20] using the Knott-Haley regression method for the binary trait sex, and the imputation method for continuous morphological traits. Other methods of analysis (EM, MR) gave similar results. Genome wide scans for QTL were performed with and without sex as an additive covariate; there was no evidence that sex acted as an interactive covariate. Genome-wide thresholds for evaluating the significance of QTL for different traits were determined from 1,000 replicated datasets from each of the scans that were performed [21]. The resulting threshold values ranged from LOD 2.70–2.81 for probability of α = 0.50, and thus a common threshold of 2.75 was used to evaluate the significance of QTL for all traits.

### Data Archiving

QTL data are available via Sal-Site (at http://www. ambystoma.org/downloads/AxAn1genotypes.txt), a public website that archives DNA sequence data, microarray data and comparative mapping data available to the Ambystoma research community.

## Results

### Effect of Sex and Tail Morphology on Regenerative Outgrowth

**Figure 1:**
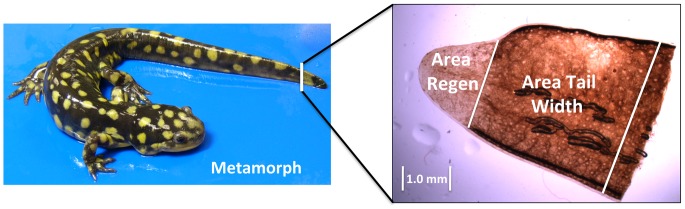
A representative tail tip that was sampled during the process of regeneration. The picture shows areas that were measured proximal and distal to the amputation plane.

We published recently that AxAn1 salamanders exhibited sexually dimorphic traits after completing metamorphosis [18]. On average, metamorphic males and females did not show significantly different snout-vent-lengths (SVL) at 300 dpf, but males presented significantly longer tails. Here, we took 217 of these post-metamorphic AxAn1 salamanders, including 110 males and 107 females, and amputated 10% of the total tail length (measured from posterior vent to tail tip, approximately 1.0–1.2 cM). The salamanders were then reared for 51 days and the tip of the tail was amputated. The amount of tail removed was 1 cm proximal to the first amputation plane. Thus, each tail tip provided an estimate of the area of the tail at the plane of amputation (tail width), and also an estimate of the amount of tissue outgrowth (tail outgrowth) (Figure 1). After an additional 46 days of growth, tail tips were collected again to provide replicate samples for each salamander.

**Figure 2:**
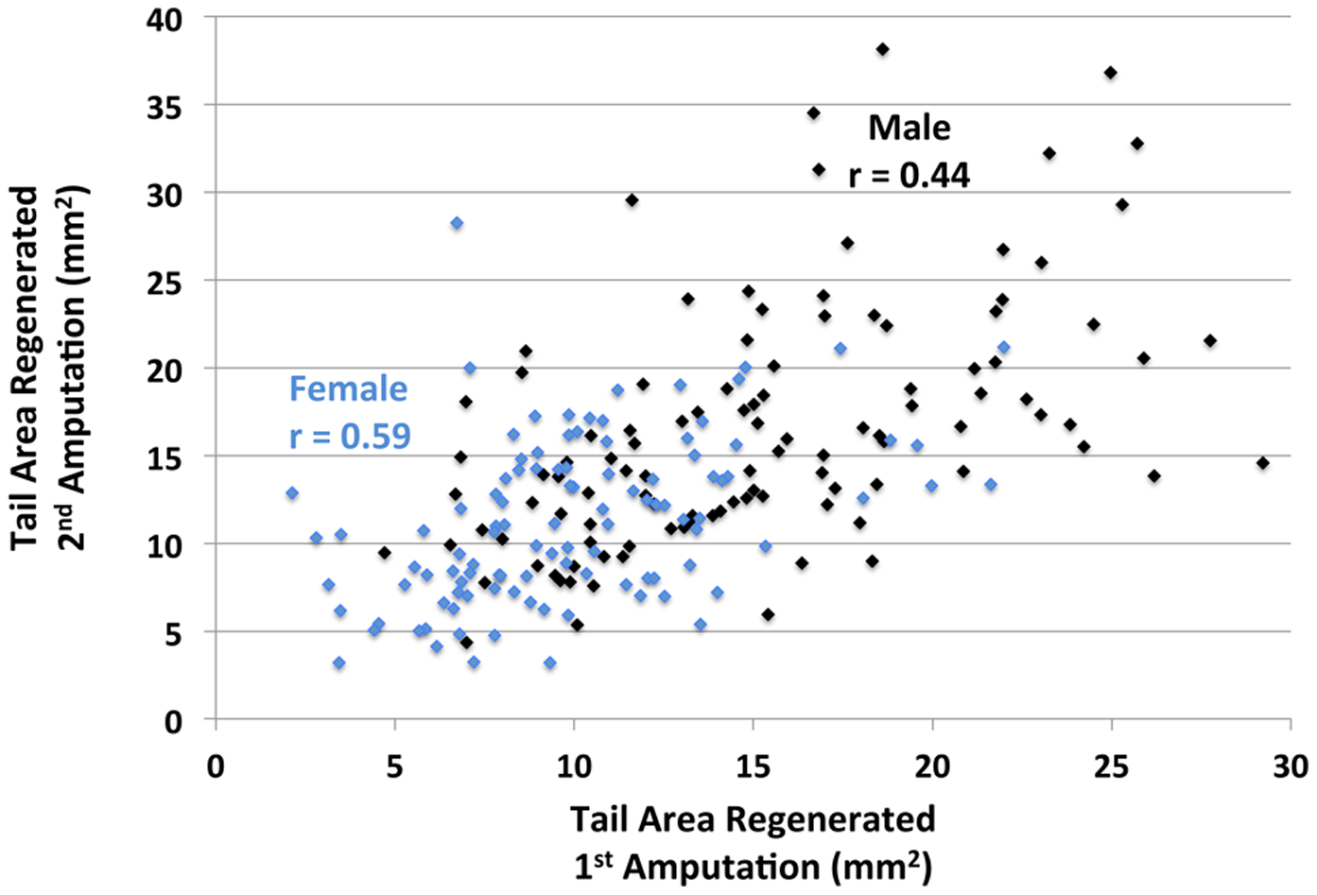
Plot showing the amount of tail tissue regenerated after the first (300 days post fertilization - dpf) and second (351 dpf) amputations. Spearman’s correlation coefficients (r) are shown for males and females.

**Figure 3:**
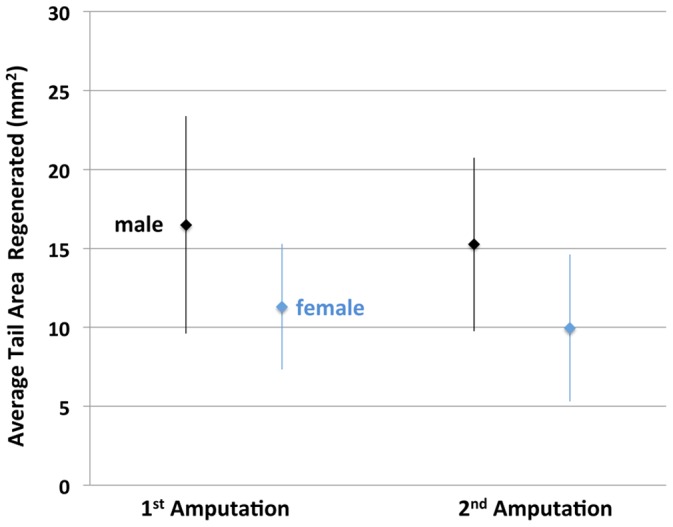
Plot showing the average amount of tail tissue regenerated after the first (300 dpf) and second (351 dpf) tail amputations for males and females. The error bars are standard deviations.

**Figure 4:**
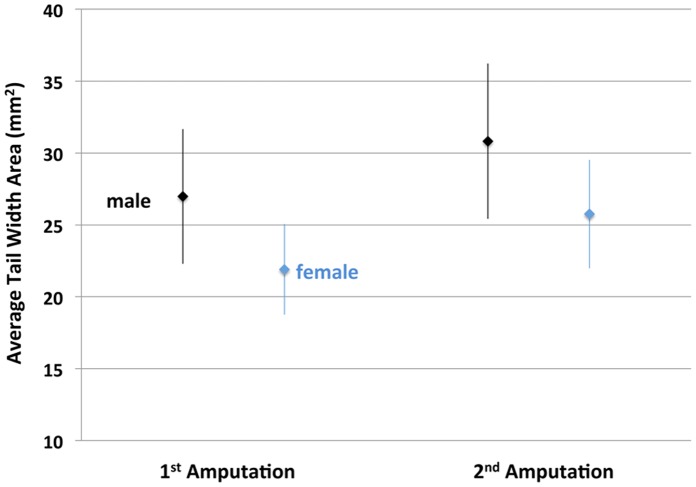
Plot showing the average width of tail area proximal to the amputation plane after the first (300 dpf) and second (351 dpf) tail amputations for males and females. The error bars are standard deviations.

We found that the amount of tail outgrowth was repeatable across times for individuals and positively correlated among males and females (Figure 2). Tail outgrowth and tail width were greater in males than females (Figures 3,4), although there was considerable overlap in these measures among individuals of opposite sex. Tail width and tail outgrowth were highly positively correlated for both episodes of regeneration (females, r = 0.79, 0.83; males, r = 0.71, 0.72). These correlations suggested that variation in tail outgrowth is more a function of tail morphology than a direct effect of genetic sex. Indeed, when the effects of sex, tail length, tail width, SVL and interaction terms were analyzed by regression modeling, only tail width explained a significant amount of variation in regenerative outgrowth (Tables 1 and 2). When treated as single variables in regression models, tail width explained 68% (p<0.001; DF = 1, 215; F = 449.39) and 66% (p<0.001; DF = 1, 214; F = 423.25) of the variation in regenerative outgrowth for the two episodes of regeneration, respectively. Overall, the results show that sex explains variation in tail morphology, however sex does not directly explain variation in tail outgrowth.

**Table 1:** Significance tests for variables that were modeled by linear regression to explain variation in tail outgrowth for the 300−351 dpf period of regeneration.

Tail Regeneration: 300–351 dpf				
Source	DF	Sum of Squares	F Ratio	Prob>F
Sex	1	30.75	3.30	0.071
TW	1	812.80	87.29	<0.0001
TL	1	1.15	0.12	0.726
SVL	1	3.17	0.05	0.816
Sex×TW	1	0.61	0.07	0.798
Sex×TL	1	22.14	2.38	0.125
Sex×SVL	1	1.85	0.20	0.656
TW×TL	1	0.51	0.05	0.816
TW×SVL	1	15.46	1.66	0.199
TL×SVL	1	5.52	0.59	0.442
Sex×TW×TL	1	14.10	1.51	0.220
Sex×TW×SVL	1	6.95	0.75	0.389
Sex×TL×SVL	1	1.16	0.12	0.725
TW×TL×SVL	1	11.82	0.12	0.725
Sex×TW×TLx SVL	1	0.39	0.04	0.838
Total	199	8411.77	25.52	<0.0001

Tail Width = TW, Tail Length = TL. The totals at the bottom of the table are for the full regression model.

**Table 2:** Significance tests for variables that were modeled by linear regression to explain variation in tail outgrowth for the 351−400 dpf period of regeneration.

Tail Regeneration: 351–396 dpf				
Source	DF	Sum of Squares	F Ratio	Prob>F
Sex	1	7.11	0.47	0.490
TW	1	1769.03	119.21	<0.0001
TL	1	13.72	0.92	0.338
SVL	1	1.20	0.08	0.777
Sex×TW	1	7.05	0.48	0.491
Sex×TL	1	33.13	2.23	0.137
Sex×SVL	1	2.37	0.16	0.690
TW×TL	1	11.94	0.80	0.371
TW×SVL	1	13.05	0.88	0.350
TL×SVL	1	0.15	0.01	0.921
Sex×TW×TL	1	6.37	0.43	0.513
Sex×TW×SVL	1	10.09	0.68	0.411
Sex×TL×SVL	1	15.80	1.06	0.304
TW×TL×SVL	1	22.66	1.53	0.218
Sex×TW×TLx SVL	1	10.68	0.72	0.397
Total	199	6091.88	31.35	<0.001

Tail Width = TW, Tail Length = TL. The totals at the bottom of the table are for the full regression model.

### QTL Analysis of Tail Morphology and Regeneration

**Figure 5:**
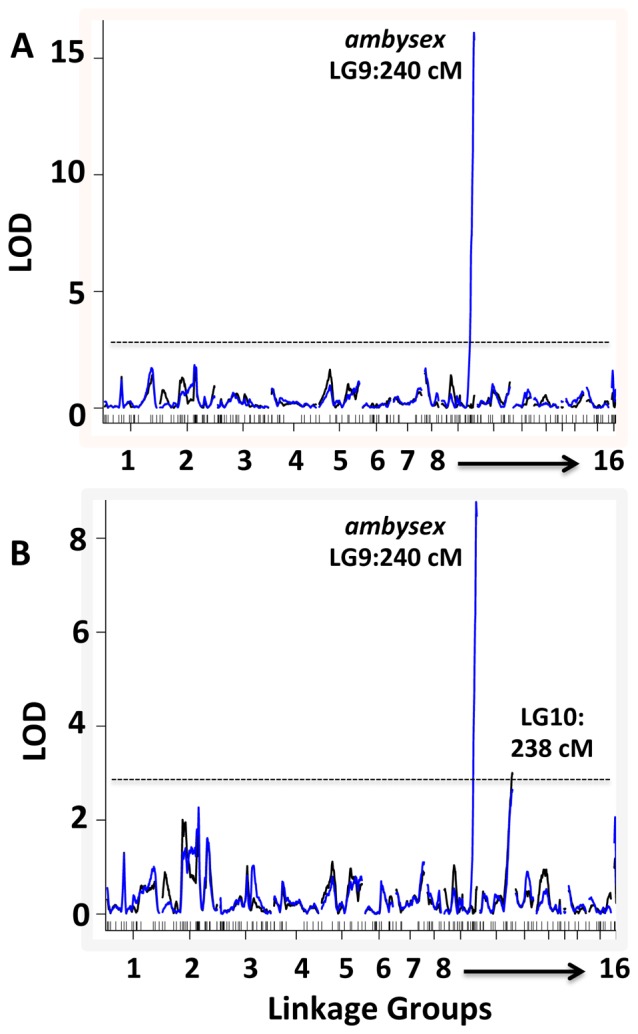
LOD plots of QTL scans for tail width (A) and tail outgrowth (B) at 351 dpf. Markers defining 16 ambystomatid linkage groups are concatenated on the x-axis. The black lines reference QTL scans after the effect of sex was removed by treating the sex-determining locus (*ambysex*) as an additive covariate. Horizontal dashed lines show QTL significance thresholds (p = 0.05).

A genetic map was created for the AxAn1 cross by genotyping 187 molecular markers that are distributed among 16 linkage groups (LG) of the *Ambystoma* genome [22]. Genome wide scans were performed to identify the most likely position of the *Ambystoma* sex-determining locus (*ambysex*) and QTL for tail width and tail outgrowth. A single highly significant LOD peak located the sex-determining locus to the end of LG 9∶240 centi-Morgans (cM) (LOD = 76.11); this map position agreed with a previous study that mapped *ambysex* using *A. mexicanum* and *A. tigrinum tigrinum* (Smith et al., 2009). QTL scans for tail width and tail outgrowth also identified *ambysex* as significant (Figure 5). When *ambysex* was treated as a covariate in genome scans of these traits, only a single QTL for tail outgrowth at 351 dpf was identified as significant (LG10∶238 cM, LOD = 3.06). This QTL mapped near *xrcc6bp1* (241 cM); at the position of this marker, 4% of the variance in tail outgrowth is explained. While it is possible that a QTL might only be detected for one episode of regeneration, without replication it is difficult to reject the possibility that small effect QTL are false positives.

While replication does not necessarily prove a QTL to be real, it increases confidence because the probability of identifying the same false-positive QTL between two episodes of regeneration is expected to be extremely low. We observed that several regions of the linkage map yielded moderate LOD scores (approximately 1.5–2.2). Given the relatively small number of meiosis available in our study, and thus limited power to detect QTL, we suspected these moderate LOD scores might implicate additional small effect, regeneration QTL. Using Student’s t-test to contrast genotypic means, we identified molecular markers from six different linkage groups that significantly affected the amount of tail outgrowth for both episodes of regeneration (Table 3). We note that all but two (*upk1b, tmem5*) of the identified markers in Table 3 also yielded significant t-statistics (p<0.05) when contrasting molecular marker genotypes for variation in tail width. The set of molecular markers from LG2, LG9, and LG16 are linked within their respective linkage groups. The LG2 markers map to the genomic location of *met1*, a QTL that was previously shown to affect metamorphic timing in this same hybrid backcross [18]. On average, tail width and tail outgrowth for both episodes of regeneration were significantly greater for *met1* genotypes that significantly delayed metamorphic timing. The LG9 markers map to the location of *ambysex,* which as we described above explains the majority of genetic variation in tail width. The LG16 markers locate to the end of the linkage group and include *tenacin C* (*tnc)*, a gene that is differentially expressed during spinal cord regeneration in salamanders [23].

**Table 3:** Molecular markers wherein genotypic means differed significantly for regenerative outgrowth between 300−351 (1^st^) and 351−400 days post fertilization (dpf) (2^nd^).

Marker ID	Map Position	Probability1^st^ 2^nd^
*sept10_b*	LG1∶ 343	0.030 0.011
*lrrc61*	LG2∶ 257	0.008 0.030
*c11orf51*	LG2∶ 261	0.008 0.012
*scap*	LG2∶ 263	0.007 0.047
*tbrg4*	LG2∶ 270	0.007 0.013
*klhl18*	LG2∶ 270	0.010 0.019
*ngfr*	LG2∶ 272	0.010 0.024
*upk1b*	LG7∶ 208	0.035 0.023
*wnt10a*	LG9∶ 214	0.004 1E-5
*accn4*	LG9∶ 217	6E-5 10E-8
*dnpep*	LG9∶ 220	2E-5 1E-8
*dnajb2*	LG9∶ 223	1E-5 1E-9
*e47c10*	LG9∶ 232	4E-7 1E-8
*ambysex*	LG9∶ 240	2E-9 3E-14
*znf776*	LG9∶ 243	2E-6 10E-10
*tmem5*	LG10∶ 230	0.002 0.034
*e11e11*	LG16∶ 0.0	0.009 0.009
*lmo4*	LG16∶ 10	0.007 0.003
*tnc*	LG16∶ 27	0.004 0.003

## Discussion

In this study, we performed the first quantitative genetic analysis of tail morphology and regeneration using a salamander model. We made an interspecific backcross (*A. mexicanum/A.* andersoni×*A. mexicanum*) and reared individuals under conditions that promoted metamorphosis into terrestrial adults. When the majority of individuals had completed metamorphosis, we measured the width and length of tails. We found that tail morphology varied greatly among individuals. The genetic factor that explained most of the variation in tail morphology was *ambysex*, the sex-determining locus on LG9 [24]. On average, longer and wider tails were associated with males while females tended to have shorter and narrower tails. Small-effect QTL were also identified for tail width and one of these located to the position of *met1,* a previously identified metamorphic timing QTL [17], [18]. Individuals that metamorphosed later in our experiment had a shorter, tail-remodeling period prior to tail amputation. As a result, these individuals had tails that were wider and morphologically more similar to the keeled shaped tails of aquatic juveniles. Other small effect QTL that were identified may contribute to differences in tail morphology between these species, as tail shape differs between *A. andersoni* and *A. mexicanum* (Voss, unpublished data). Overall, we found that variation in adult tail morphology was associated with genetic factors that regulate sex differentiation, metamorphic timing, and species-specific differences in tail morphology.

The primary objective of our study was to search for genetic factors that explain variation in tail regeneration. After metamorphosis, we amputated tails of all adults at the same anatomical position, and then after several weeks, measured the amount of tissue that regenerated. We then repeated the amputation and measurement procedure to obtain replicate data for all individuals. Although we found that regenerative outgrowth was precise among individuals that presented morphologically variable tails, we could only attribute a small amount of the variation in regenerative outgrowth to QTL. Instead, we found that approximately 66–68% of the variation in regenerative outgrowth was explained by the width of the tail at the amputation plane. These results show that variation in regenerative outgrowth is indirectly associated with the actions of genes that cause variation in tail morphology.

QTL studies of tissue regeneration in animals have only been performed using the MRL mouse model of wound healing. In these studies, multiple QTL were identified that collectively explain as much as 70% of the variation in wound healing, and the same 2 QTL were identified as significant between independent studies [6], [7]. Blankenhorn et al. [9] found that genetic sex affected healing, with certain QTL significant for only males or females, and females healing faster and more completely than males. Much like the findings of our study, indirect genetic effects on tissue regeneration were also identified, with body weight and healing ability in males attributable to the same QTL. The QTLs identified from the MRL mouse do not suggest potential candidate genes for QTL identified in our study; this is not surprising because wound healing represents a brief, initial phase of tail regeneration, and during this phase there is no regenerative outgrowth of tissue.

Although salamander tail regeneration has been studied for almost 300 years, many fundamental questions remain unanswered. Our results allow us to address an outstanding question concerning variation in regeneration along the rostral-caudal tail axis. In newts, the rate of regenerative outgrowth is greater if the tail is amputated farther from the tail tip [14]. For example, if salamanders had their tails amputated 1 or 2 cM from the tail tip, on average more regenerative outgrowth per unit time would be observed for the more rostral amputation. While this suggests that the position of amputation along the tail axis determines the amount of regenerative outgrowth, salamander tails are somewhat conical in shape; size and shape parameters (e.g. thickness, width and diameter) decrease in a rostral-caudal direction. Because spatial coordinates of the tail axis co-vary with tail shape, it is not clear if regenerative outgrowth varies as a function of position along the rostral-caudal axis or tail shape. In our experiment, we controlled for the position of amputation – we amputated all salamander tails at the same relative position of the rostral caudal axis. Our results show that genetically based differences in tail width, measured 4 mm from the amputation plane, largely explain variation in regenerative outgrowth. This suggests that regenerative outgrowth is regulated locally by factors that scale with tail width. Because tail regenerative outgrowth scales exponentially as a function of tail diameter [15], we suspect factors that are also exponentially distributed, increasing in a caudal-to-rostral direction. This implicates many components of the tail because cell numbers, tissue biomass (e.g. vasculature, innervating axons, muscle, cartilage/bone), and extracellular molecules are all expected to vary exponentially along the axis of a conically shaped, three-dimensional tail. Additional studies are needed to investigate the quantitative effects of cells, tissues, and molecules on regenerative outgrowth.

We conclude by noting a practical finding of our study for scientists that use ambystomatid salamanders as research models. Juvenile ambystomatid salamanders are commonly used in studies of regeneration. The sex of juvenile salamanders is particularly difficult to determine and quite unreliable, as even necropsies require mostly-differentiated testes or ovaries in order to successfully assign sex to an individual. Without genetic markers for determining the sex of a salamander [24], individuals must be reared for many months in the lab, until they show sexually dimorphic characteristics. In our study, we did not find an effect of sex on regenerative outgrowth for individuals undergoing sexual differentiation and post-metamorphic tissue remodeling. Our results show that sex and maturation only affect regenerative outgrowth indirectly, by affecting variation in tail morphology. Thus, as long as variation in tail morphology is considered as a source of variation in studies of regenerative outgrowth using ambystomatids, it is not necessary to know the genetic sex or stage of maturation.
